# Management of Medico-Legal Risks in Digital Health Era: A Scoping Review

**DOI:** 10.3389/fmed.2021.821756

**Published:** 2022-01-11

**Authors:** Antonio Oliva, Simone Grassi, Giuseppe Vetrugno, Riccardo Rossi, Gabriele Della Morte, Vilma Pinchi, Matteo Caputo

**Affiliations:** ^1^Legal Medicine, Department of Health Surveillance and Bioethics, Università Cattolica del Sacro Cuore, Rome, Italy; ^2^Risk Management Unit, Fondazione Policlinico A. Gemelli Istituto di Ricovero e Cura a Carattere Scientifico, Rome, Italy; ^3^International Law, Institute of International Studies, Università Cattolica del Sacro Cuore, Milan, Italy; ^4^Department of Health Sciences, Section of Forensic Medical Sciences, University of Florence, Florence, Italy; ^5^Criminal Law, Department of Juridical Science, Università Cattolica del Sacro Cuore, Milan, Italy

**Keywords:** privacy, artificial intelligence, big data, risk management, COVID-19

## Abstract

Artificial intelligence needs big data to develop reliable predictions. Therefore, storing and processing health data is essential for the new diagnostic and decisional technologies but, at the same time, represents a risk for privacy protection. This scoping review is aimed at underlying the medico-legal and ethical implications of the main artificial intelligence applications to healthcare, also focusing on the issues of the COVID-19 era. Starting from a summary of the United States (US) and European Union (EU) regulatory frameworks, the current medico-legal and ethical challenges are discussed in general terms before focusing on the specific issues regarding informed consent, medical malpractice/cognitive biases, automation and interconnectedness of medical devices, diagnostic algorithms and telemedicine. We aim at underlying that education of physicians on the management of this (new) kind of clinical risks can enhance compliance with regulations and avoid legal risks for the healthcare professionals and institutions.

## Introduction

Digital revolution is changing and will radically change the way healthcare is conceived (1). Currently, several artificial intelligence (AI) products have been developed, covering all aspects of healthcare, like the prediction of the risk of acute or chronic disease (e.g., cardiovascular risk, gastrointestinal bleeding, glaucoma), the prediction of risk of cancer/cancer recurrence and the survival likelihood in oncologic patients, the management of common chronic conditions (e.g., optimization of insulin dose in type-1 diabetes), the organization of clinical, surgical and anesthesiologic services, and the discovery of new drugs (2–13). AI can work and evolve only if personal health information is collected in datasets. Currently, in healthcare enormous amounts of data are normally collected—not only descriptive information (e.g., name, occupation, physical and mental conditions, genetic profile) but also data acquired by ambient sensors, images (obtained through endoscopy, radiologic techniques or dermoscopic mapping) and molecular/genetic data (5, 8, 14–16). Moreover, there are portable/wearable/implanted medical and non-medical devices that regularly collect data that can be used for predictions useful for preserving and improving the health of both individuals and the entire community (17). Health and genetic data are the most sensible personal information and their misuse can be extremely harmful and discriminatory. Globally, there are different regulatory approaches intended for privacy protection, but the European Union (EU) regulatory framework is often considered the broadest (6, 18). Moreover, in Europe privacy protection is a right guaranteed both by the European Charter of Human Rights and by some national constitutions (for example in Spain) (6, 18). However, from a legal point of view privacy is never an “absolute” right, but it has many trade-offs, that must always be carefully considered to decide what right should prevail in the specific situation (16).

This scoping review is aimed at underlying the medico-legal and ethical implications of the main artificial intelligence applications to healthcare. Starting from a summary of the United States (US) and European Union (EU) regulatory frameworks, the current medico-legal and ethical challenges are discussed in general terms before focusing on the specific issues regarding informed consent, errors/cognitive biases, diagnostic algorithms and telemedicine.

## Methods

The review question was “what are the main medico-legal and ethical issues of general interest concerning artificial intelligence applied to healthcare?” Since the question is very broad, the targets are very diverse and the aim is to describe an overview of the available research evidence, a systematic review approach was not chosen. Two investigators searched published studies through the electronic database MEDLINE via PubMed. They combined three classes of search terms (the classes were connected through the Boolean operator AND, while the items of each class were combined through the Boolean operator OR): (1) artificial intelligence, algorithms, personal data processing; (2) COVID-19, informed consent, medical malpractice, cognitive bias, automation, interconnectedness, robot, telemedicine; (3) medico-legal issues, ethical issues, medico-legal implications, ethical implications, medico-legal risks. The eligibility criteria were the language (only papers written in English were considered), the publication date (between January 1, 2015 and June 30, 2021) and the publications status (only papers that had been fully published online were selected). Search was not filtered by article type. The 41 papers considered for the review were selected on the basis of their relevance according to the review question.

## US and EU Regulatory Frameworks

In the US, Table 1 AI products must be approved by the Food and Drug Administration (FDA, that classifies them as “software as a medical device”), while the collection, storage and disclosure of personal health information is regulated mainly by the 1996 Health Insurance Portability and Accountability Act (HIPAA) (19). Personal health information (individually identifiable health information, a category that also includes genetic data) can be lawfully disclosed, for example, to the individual upon his legitimate request or to public authorities (e.g., if the disclosure is allowed/required by an applicable law, for public health activities and purposes, and for judicial and administrative proceedings). In general, when individually identifiable health information is used or disclosed, the minimum necessary standard must be adopted. A HIPAA authorization written in plain language (patient consent) is generally required, for example, for the use or disclosure of psychotherapy notes or of personal health information for research or marketing purposes. In most of the circumstances, the patient can restrict or prohibit some or all of the uses or disclosures of his personal health data (when he can't express his will - for example, because of emergency conditions - the disclosure of his data can be allowed if it is consistent with his prior expressed preference and pursues his best interests). However, HIPAA regards only individually identifiable health information and covered entities (healthcare provides, health plans—like health insurance companies—and healthcare clearinghouses). Therefore, this law doesn't apply to deidentified data (that can be freely used, for example, for research) and non-covered entities (e.g., private firms) (16, 20). In other words, most health apps are not covered by HIPAA (21). Deidentification is an articulate process: HIPAA sets a list of 18 personal identifiers (e.g., name, address, medical record number) that must be removed for the lawful use/disclosure of data (safe harbor method) (16). Alternatively, an expert must assess a very small risk of re-identification applying a rigorous and transparent scientific and statistical methodology. Finally, in the US a broader protection of anonymized data can be given by statal laws, like the California Consumer Privacy Act (defined the “little sister of GDPR”), that covers also data that can be indirectly identified (e.g., through IP address) (21, 22).

**Table 1:** Main differences between US and EU regulations regarding data processing.

	**HIPAA privacy rule**	**General data protection regulation**
Country	United States	European Union
Protected data	Protected health information = individually identifiable health information	Personal data = any information (physical, physiological, genetic, mental, economic, cultural or social data) relating to an identified or identifiable natural person
Covered entities	Health plans, clearinghouses, health care providers (and their business associates)	Companies and entities which process personal data as part of their activities
De-identification methods	Assessment of a very small risk of re-identification performed by an expert or reversible (e.g., encoded)/irreversible removal of 18 identifiers (like name, personal dates and biometric identifiers) [45 CFR Subtitle A (10–1–02 Edition) § 164.514]	[Recommended] anonymization (irreversible removal of personal identifiers) or pseudonymization (reversible removal of personal identifiers) [Art. 9, Art. 89]

EU has several peculiarities from a legal point of view. First, there is a significant heterogeneity among EU countries in terms of digital health funding, readiness and use (23). Moreover, in EU, there is no common regulatory framework for medical liability, since, despite some common legal principles, there are enormous legal differences among the juridical cultures of the Member States (e.g., in Italy medical errors can also be criminally persecuted) (24). That being said, privacy protection is guaranteed by the General Data Protection Regulation (GDPR), that applies when personal data are processed by a processor or controller in the context of the activities of its establishment in EU. GDPR is usually considered broader than US laws (16, 20). In particular, the GDPR definition of health data is extensive, regarding even the data that can reveal the health status or risk of patient only if combined with other information (25). Data can be processed lawfully and in a transparent manner only for “specified, explicit and legitimate purposes” and must be “adequate, relevant and limited to what is necessary in relation to the purposes for which they are processed” (principle of minimization) (Art. 5). Data can be stored for longer period than that necessary for the purposes of their processing only under particular circumstances (e.g., archiving purposes in public interest or scientific research), and the controller is always accountable for their protection (Art. 5). The explicit consent of the data subject can be waived, for example, for compliance with a legal obligation, for reasons of substantial public interest or for scientific research. In this latter case, for instance, according to articles 9 and 89 of the GDPR, there must be appropriate safeguards (specified by Union or Member State laws), among which the GDPR indicates a reversible form of anonymization called pseudonymisation. However, up to date many Member States have not approved laws of this kind yet (26). Moreover, Malgieri et al. underlined a significant disparity between private and public universities in terms of legal/ethical standards for data processing, since private institutions must comply with much stricter criteria (they must prove the so-called “legitimate interest”) (26). Regarding international law aspects, a US health institution, physician or geneticist could be liable under GDPR if its/his patients are EU citizens (16).

## Current Medico-Legal and Ethical Challenges

Grande et al. identified five medico-legal and ethical issues regarding personal data processing: invisibility (patients don't know how their data are processed), inaccuracy of the collected data, immortality (no timeline for the data storage), marketability and identifiability (even when data are anonymized, it is often possible to reidentify the patient) (27). These issues concern both the data that the patients agree to send to the provider of a service and those that are involuntarily left as “digital health footprints” when a digital device is used (27). From a medico-legal perspective, the main risk is that of re-identification (16). Indeed, personal health information can be used for unlawful purposes (for instance, a genetic predisposition to a disease can be used to increase the cost of the insurance coverage) or to obtain more sensible information (e.g., some genetic markers can be used to predict externally visible characteristics of the individual like the skin tone and the color of the eyes).

Data protection has become even more critical since the beginning of the pandemic: the processing of big data was (and is) used to enhance the COVID-19 control measures (e.g., through contact tracing, risk prediction algorithms), adopting two different approaches: some nations adopted the data-first approach (in which storage and communication to health and research institutions of the data represent the priority) and some others chose the privacy-first approach (in which health authorities do not know individual movements and interactions) (28, 29). In any case, each country is storing an unprecedented amount of population data of every kind (e.g., health data, individual movements and interactions), that, if not properly processed, could lead to catastrophic outcomes (28). Therefore, cybersecurity should still represent a priority. Morley et al. observed that an application for tracking and tracing of COVID-19 cases can be considered ethically justifiable only if it complies with “high-level principles” (necessity, proportionality, scientific soundness, and time-boundedness) and “enabling factors” (e.g., the use of the application is voluntary, a consent is requested, stored data can be erased upon users request, its purpose is defined and limited) (30).

Besides the risk of data misuse, AI systems are vulnerable to both software and hardware faults, that can be extremely harmful for patients. For instance, inadequate training data or wrong design choices can cause abnormal system behavior (31). These errors can be due to the users rather than to the developers: for instance, an AI system can make wrong (and potentially harmful) decisions if it is not used in the original design context (31). Moreover, logic, memory or communication components of the devices can be affected by permanent or transient hardware failures (like the transient failures—also called “soft errors”—represented by bit flips due to radiation particles) (31).

For these reasons, AI products designed for healthcare are considered by EU Commission as “high-risk” and, before they can be put on the market, have to meet these requirements: adequate risk assessment and mitigation systems, high-quality datasets, logging of activity, detailed documentation to prove the compliance with legal requirements, clear and adequate information to the user, appropriate human oversight measures and high level of robustness, security and accuracy (https://ec.europa.eu/commission/presscorner/detail/en/IP_21_1682).

## Consent

AI can also be used to do clerical work on behalf of physicians, who then would have more time to communicate to patients (6). In digital health, the central role of communication and informed consent is threatened by the fact that artificial intelligence softwares are often not transparent. This is a serious legal issue, since patients should give their consent without fully understanding how their data will be processed. Moreover, many patients lack the level of technological literacy necessary for fully understanding the pros and the cons of digital health (32). This issue undermines the patients' engagement and can impede to obtain affirmative, unambiguous, and conscious decisions required in current medicine (32). On the other side, the access to some data (e.g., glucose blood levels reported by a microinfusor) can empower patients with a more direct and efficacious role in prevention (e.g., the diabetic person could better understand what behavior increases the glucose blood levels) and can reduce the information asymmetry between them and physicians/geneticists. Empowering patients would also mean to reduce the uncertainty regarding the causes of adverse outcomes: if a device can record all the inputs and the outputs, this “black box” can be used for both improving patient education and, after an adverse event occurred, reconstruct what was its cause. In this way, physicians, healthcare institutions and device manufacturers could not be considered wrongly liable for adverse events who the patients mainly caused (e.g., if a poor diabetes control is proved to be due to an abnormal intake of food). Regarding consent, specific principles must be applied to children. Generally, the relatives are entitled for giving consent to process data of their siblings, but the minor should be still heard (this principle has been expressed, among the others, by United Nations General Assembly in 1989 and in some countries, like Italy, it is regulated by national laws). Moreover, when data of a minor must be processed, the proof that the best interests of the child are pursued is needed to go forward (in Europe this duty is guaranteed by the 2007 European Convention of Human Rights, the 2012 Charter of Fundamental Rights of the European Union and the 2013 United Nations Committee on the Rights of the Child) (33).

## Cognitive Bias and Risk of Medical Malpractice

AI can strongly influence healthcare professionals, changing the approach to their profession. In particular, AI is associated with the risk of deskilling (the physician outsources his tasks to the software, losing his technical and non-technical skills) and overfaith (the physician relies on the results obtained by the algorithm, not critically evaluating/considering the possibility of errors) (6, 34–36). Passively accepting AI outputs is called automation bias and represents a source of important medico-legal risks, since AI can be wrong (because of an operation error or of an operation on wrong data). For instance, Bond et al. found that the diagnostic accuracy of the interpreters of ECGs, especially if not specialized in cardiology, nearly halved when the automated diagnosis software missed the correct diagnosis (37). Adopting the proper approach to AI, the physicians would not lose their technical skills - but their skills could improve on one side and worsen on another side. For instance, Carter et al. observed that if normal mammograms are triaged out by AI radiologists would improve their skills in interpreting pathological images but could lose their skill to recognize normal images (36). For these reasons, proper and updated medical education is needed: for instance, in Italy less than a fourth of young physicians has a proper knowledge of artificial intelligence and big data, and this could be cause scarce engagement and higher risk of deskilling (38). However, even in case of proper education, the issue of the interpretability/explainability of AI results remains. Some authors distinguish two models of interpretability (i.e., the interpretation of the general behavior of the AI system) from inference interpretability (i.e., the interpretation of the instance-specific decisional process of the AI system), but in any case physicians/geneticists can't be out of the loop and must always be able to explain and—in case of adverse outcome—justify the logical process behind a diagnosis or a treatment choice (11, 32). Indeed, as observed by Reddy, “trust in clinicians encompasses trust in the clinical tools they choose to use, and in the selection of those tools, including AI-based tools” (19). The first issue regarding interpretability and transparence of the process is represented by the fact that the outputs of an algorithms and the algorithms themselves are often a “black box” (5, 6). In particular, artificial intelligence produces a prediction but cannot explain its results and it is not capable of causal inference. For instance, Verghese et al. reported the case of an algorithm developed for crime forecasting that assigned a significantly higher risk of reoffence to black individuals than to white persons without a clear statistical reason (39). Many of the most accurate algorithms are not particularly transparent, and this could create a trade-off between accuracy, intellectual property protection and explainability (36, 40). A second issue is represented by the fact that the direct output of AI tools is often represented by raw results (e.g., those produced by an implantable cardiac defibrillator), that can be hardly understandable for both patients and physicians (11). Finally, artificial intelligence can be adaptive, evolving through a process of continual learning, and AI devices can autoupdate (19, 41). Rapid regulatory obsolescence is a critical issue, since it can create regulatory gaps that can represent a serious hazard for data protection (40).

## Medico-Legal Issues of Diagnostic Algorithms

Machine learning is a technology that, working on a dataset (training data), can develop predictions through algorithms (a process also known as “generalization”). It is modeled on human brain and can operate through supervised or unsupervised algorithms (6, 42). Supervised algorithms identify patterns in well-organized databases, in which each entry is correctly labeled (43). Supervised algorithms are subdivided into classification and regression algorithms. The latter work on continuous data and are aimed at reliably predicting an output variable, while classification algorithms process discrete data and divide the dataset into different classes, predicting to which class an input variable belongs. On the other side, unsupervised algorithms try to deduce a “natural” pattern evaluating the relationships among unlabeled data (for example, through the individuation of similarities and differences among data) (6, 43). Sometimes, the term “semi-supervised” is used to define the algorithms that use incomplete input information (44, 45). Unsupervised algorithms are complex and mainly used for data mining (44). Currently, the on-demand access to graphical processing units technology needed to process data is also provided by cloud-computing platforms, and thus softwares for health/genetic data can be relatively easy to use (43).

Quantity and quality of data are core factors for AI and are factors of great medico-legal interest (because a low-quality algorithm must be considered unreliable and thus cannot be adopted). Indeed, when there are few data (and/or the algorithm is too much complex), there is the risk of overfitting: the prediction is valid for the dataset but it could prove to be unreliable if other data are added (43, 46). There are some strategies to reduce the risk of overfitting (e.g., metaanalyses of different algorithms applied to the same dataset; data augmentation: for example, considering an image from different perspectives in order to obtain more data from the same image) (43). The big quantity of data (big data) needed for AI to properly function is frequently expressed with the term “data hungriness” (11). Data hungriness is related to substantial medico-legal issues, since a single institution often does not have enough data to develop reliable predictions and in complex (multifactorial) diseases (like cancer) it is frequently necessary to combine more kinds of big data (e.g., familiarity, behavior, diet, genetic profile) coming from different sources (47). Therefore, data are frequently transferred and shared. Over the last years several EU and non-EU countries transferred large amounts of deidentified personal health information to private companies (in order to develop AI softwares) (36). In these cases, the main legal problem is the risk of re-identification of anonymized data, an operation that can be performed both by hackers and the private companies that receive the big data (36, 48). This issue is particularly important if it is considered that many producers of health-related AI (e.g., Google) also detain many non-health data that could be used in combination to re-identify the specific individuals (36). Therefore, data transfer, even when it occurs legally, can still represent a serious risk for privacy.

## Medico-Legal Issues Linked to Automation and Interconnectedness

As said, when most of the crucial decisions are made by AI, especially when AI is not transparent and when there are multiple interconnected devices, causal inference probably is the main issue from a medico-legal point of view (49). Indeed, in these cases, causal processes are often very complex and the provability of individual responsibility is often difficult. The commonest liability rule is the so-called negligence (or fault-based) liability: the plaintiff is compensated if his damage ant the breach of a duty are proven and the responsible entities are identified (46). In 2021, Zech noted that strict liability can be more adequate for errors committed by AI systems than the negligence liability (49). Under this rule, the plaintiff is compensated simply if he proves to have been damaged (regardless of the proof of the breach of a duty). In particular, Zech underlined that social first party insurances could compensate patients without an individual attribution of responsibility (49). However, as noted by the author, incentives for risk control created by liability rules could be lost if the developers and users of AI cannot be held liable (49).

Causal inference and individual attribution of responsibility are extremely complex issues in robotic surgery: in these cases, it can be difficult to determine whether the surgeon or the software committed the error. For this reason, some authors proposed to install into the robots devices that record any input and output (similarly to flight recorders), while in EU a recent (2017) Resolution advocates creating a specific legal status for robots in order to make them liable for their errors (48). However, the error rate in robotic surgery tends to be lower than in traditional surgery (48). This fact represents a serious issue (from a medico-legal and economic point of view) in legal systems in which the compliance with best practices is mandatory (e.g., in Italy).

Robots are also linked to specific ethical issues, like: the replacement of human operators, the risk of dual use (harmful use—use for warfare or terrorism—of AI systems developed for civilian purposes), the anthropomorphisation of the robots (that can cause social and psychological issues to the users), the social and ethnic gaps (a fair and equal access to new technologies) and the environmental impact of robots (50, 51). At this regard, Campanozzi et al. underlined the importance of building trust in social robotics: developing acceptable and sustainable robots that meet people's needs, values and attitudes, adverse events due to overtrust or undetrust in AI products could be avoided (52).

## Medico-Legal Issues of Telemedicine

Telemedicine is having a significant impact on healthcare services like preventive medicine and follow-up of chronic conditions (24). In general, it is considered beneficial for both the healthcare institutions (that can offer their services also to distant people or to elder/physically impaired/sedentary persons who normally don't go to hospitals for non-urgent conditions) and for the patients (who can save more than 100 min per visit) (24, 53). The main medico-legal issue of telemedicine is the so-called de-coupling: the physician and patient are in different locations or even in different states (32). Therefore, since different states usually have different regulations, in case of claimed medical malpractice, it can be controversial what law is applicable.

A particular kind of telemedicine is mobile health: digital applications can, for instance, enhance the compliance with programs of primary or secondary prevention and permit to perform “domestic triage” (i.e., symptom checkers applications used to stratify the risk, reducing the number of avoidable hospitalizations) (18). Moreover, during COVID-19, both public and private entities developed applications for contact tracing, movements tracking, enforcing quarantine compliance and symptom checking (18, 54). The specific legal and medico-legal issues of mobile health are related to the risk of “digital health footprints,” left when a digital device is used (27). Grande et al. observed that US laws don't adequately protect patients privacy, discussing five issues regarding digital health footprints: invisibility (patients don't know how their data are processed), inaccuracy of the collected data, immortality (no timeline for the data storage), marketability and identifiability (even when data are anonymized, it is often possible to reidentify the patient through the combination of digital footprints) (27).

## Conclusion

Healthcare is radically being changed by the introduction of artificial intelligence. Despite each country has its own regulatory framework on data processing and protection, some principles are shared by Western countries, like the possibility of processing de-identified information for research even without the patient consent. Storing and processing big (health and genetic) data is the only way to develop reliable predictions in both clinical and genetic fields but, at the same time, represents a serious threat for privacy protection. Data controller can be considered accountable for data breach and/or failure to comply with regulatory standards. Therefore, since data sharing is essential to allow the full development of artificial intelligence, it is fundamental that physicians learn how to fully comply with regulations.

## Author Contributions

AO, MC, VP, and SG ideated the project. All authors contributed to the article and approved the submitted version.

## Funding

This work has been supported by Fondi di Ateneo, Linea D3.2—Project Funzioni pubbliche, controllo privato. Profili interdisciplinari sulla governance senza governo della società algoritmica Università Cattolica del Sacro Cuore.

## Conflict of Interest

The authors declare that the research was conducted in the absence of any commercial or financial relationships that could be construed as a potential conflict of interest.

## Publisher's Note

All claims expressed in this article are solely those of the authors and do not necessarily represent those of their affiliated organizations, or those of the publisher, the editors and the reviewers. Any product that may be evaluated in this article, or claim that may be made by its manufacturer, is not guaranteed or endorsed by the publisher.
